# An Analysis of 12,247 Severe Suicide Attempts Between 2010 and 2023 by Trauma-Inducing Mechanisms: Increasing Frequency and Sex-Specific Differences

**DOI:** 10.3390/jcm15062299

**Published:** 2026-03-17

**Authors:** Maximilian Leiblein, Philipp Störmann, Rolf Lefering, Ingo Marzi, Nils Wagner, the TraumaRegister DGU

**Affiliations:** 1 Department of Trauma Surgery and Orthopedics, University Hospital Frankfurt, Goethe University, 60596 Frankfurt am Main, Germany; maximilian.leiblein@unimedizin-ffm.de (M.L.); philipp.stoermann@unimedizin-ffm.de (P.S.); marzi@trauma.uni-frankfurt.de (I.M.); 2 Institute for Research in Operative Medicine (IFOM), Witten/Herdecke University, 51109 Cologne, Germany; rolf.lefering@uni-wh.de; 3 Section of Emergency, Intensive Care and Severe Injury Care (NIS), German Trauma Society (DGU), 10623 Berlin, Germany

**Keywords:** suicide attempt, trauma, registry, COVID-19, injury mechanism, mortality, prevention, sex differences

## Abstract

**Background/Objectives**: Suicide attempts represent a major global health problem. Traumatic suicide methods, such as falls from great heights, stab wounds, and gunshot wounds, frequently result in severe or fatal injuries. The COVID-19 pandemic, as well as broader societal stressors including economic uncertainty and geopolitical conflicts, has substantially increased psychological stress in the population and has been discussed as a potential influencing factor for suicidal behavior. The aim of this study was to analyze severe traumatic suicide attempts and to evaluate the potential influence of the COVID-19 pandemic in a multicenter analysis of the TraumaRegister (TR) DGU^®^. **Methods**: This retrospective multicenter analysis is based on the TraumaRegister DGU^®^, a standardized database for seriously injured patients. Patients from Germany, Austria, and Switzerland from 2010 to 2023 with an Injury Severity Score (ISS) ≥ 9, an age ≥ 10 years, and a documented suicide attempt, who arrived at the hospital alive, were included. **Results**: Among severely injured trauma patients recorded in the registry, 12,247 (4.4%) cases were classified as suspected traumatic suicide attempts. Severe traumatic suicide attempts showed a clear age-dependent distribution, with a marked increase from adolescence and a plateau between 20 and 55 years of age. Both the mean age of the general population and the age of patients with suicide attempts increased over the study period. This trend was reflected in the rise in the ≥70-year age group from 13.6% in 2010 to 19.6% in 2023. The most common method was jumping from a height greater than 3 m (65.3%), followed by stab wounds (11.9%) and gunshot wounds (8.0%). While a significant decline in severe traumatic suicide attempts was observed between 2010 and 2019, a significant increase to 4.5% occurred in 2020, remaining at a comparable level in the following years. Sex-specific differences were observed, with penetrating injuries occurring more frequently in men, whereas jumps from heights > 3 m were more common among women. The highest hospital mortality was observed in gunshot injuries (67.9%). **Conclusions**: This study demonstrates an increase in severe traumatic suicide attempts in 2020 that persisted at a similar level until 2023. Sex-specific differences in suicide methods highlight the need for targeted prevention strategies. In addition, demographic aging is reflected in the increasing proportion of suicide attempts among older individuals, emphasizing the need for age-specific prevention measures. The relatively high survival rate after certain methods, particularly after falls from height (77%), underlines the importance of structured postoperative psychiatric care pathways. These findings specifically reflect traumatic suicide attempts resulting in severe injury and requiring trauma center treatment.

## 1. Introduction

Suicide attempts represent a major global public health problem and pose substantial medical, psychological, and societal challenges. In Germany alone, 10,300 people died by suicide in 2023 [1]. Among the various methods used in suicide attempts, trauma-inducing mechanisms such as jumping from great heights and the use of firearms or sharp objects frequently lead to severe injuries or fatal outcomes.

Patients who survive traumatic suicide attempts often present with severe injuries requiring extensive medical treatment and intensive care. Previous studies have demonstrated increased injury severity and mortality in this patient group. In an analysis of falls from height, the mean Injury Severity Score (ISS) was significantly higher in suicide attempts compared with unintentional falls (31.8 vs. 26.4) [2,3]. Correspondingly, mortality was also significantly higher in the suicide group (21.4% vs. 14.2%) [2]. In addition, analyses from the German TraumaRegister DGU^®^ have demonstrated sex-specific differences in the methods used for suicide attempts [4].

The COVID-19 pandemic has been associated with increased psychological stress in many populations. The German federal government’s loneliness strategy describes a rise in perceived loneliness among young people since the pandemic [5]. However, evidence regarding the impact of the COVID-19 pandemic on suicidal behavior remains inconsistent. While overall suicide rates in Germany remained largely stable during the pandemic period [6], a significant increase in severe suicide attempts among male adolescents was reported during the second lockdown in 2021 [7]. Suicide attempts involving trauma-inducing mechanisms can result in long-term physical consequences, including reduced mobility, impairment of bladder and bowel function, and chronic pain, which may be particularly devastating for younger individuals.

Against this background, further investigation of severe traumatic suicide attempts is warranted. The aim of the present study was therefore to analyze severe suicide attempts involving trauma-inducing mechanisms among severely injured trauma patients recorded in the TraumaRegister DGU^®^. By focusing on severely injured trauma patients, this study provides insight into a clinically relevant subgroup of suicide attempts requiring trauma center care. We hypothesized that, during the COVID-19 pandemic, a greater proportion of younger patients would attempt suicide using trauma-inducing mechanisms.

## 2. Materials and Methods

The TraumaRegister DGU^®^ of the German Trauma Society (Deutsche Gesellschaft für Unfallchirurgie, DGU) was founded in 1993. The aim of this multi-center database is a pseudonymized and standardized documentation of severely injured patients. Data are collected prospectively in four consecutive time phases from the site of the accident until discharge from hospital: (A) pre-hospital phase, (B) emergency room and initial surgery, (C) intensive care unit and (D) discharge. The documentation includes detailed information on demographics, injury pattern, comorbidities, pre- and in-hospital management, course on intensive care unit, and relevant laboratory findings including data on transfusion and the outcome of each individual. The inclusion criterion is admission to hospital via the emergency room with subsequent ICU/ICM care or reaching the hospital with vital signs and dying before admission to the ICU. The infrastructure for documentation, data management, and data analysis is provided by AUC—Academy for Trauma Surgery (AUC—Akademie der Unfallchirurgie GmbH)—a company affiliated to the German Trauma Society. The scientific leadership is provided by the Committee on Emergency Medicine, Intensive Care and Trauma Management (Sektion NIS) of the German Trauma Society. The participating hospitals submit their data pseudonymized into a central database via a web-based application. Scientific data analysis is approved according to a peer review procedure laid down in the publication guideline of TraumaRegister DGU^®^.

The participating hospitals are primarily located in Germany (90%), but a rising number of hospitals of other countries contribute data as well (at the moment from Austria, Belgium, China, Finland, Luxembourg, Slovenia, Switzerland, The Netherlands, and the United Arab Emirates). Currently, more than 28,000 cases from almost 700 hospitals are entered into the database per year. Participation in TraumaRegister DGU^®^ is voluntary. For hospitals associated with TraumaNetzwerk DGU^®^, however, the entry of at least a basic dataset is obligatory for reasons of quality assurance.

The present study (TR-DGU project ID 2024-022) was reviewed and approved through the internal review process in accordance with the publication policy of the TraumaRegister DGU^®^.

The standard dataset of the TR-DGU was used for this retrospective analysis. In the TraumaRegister DGU^®^, injury events are documented in two steps. First, the presumed cause of injury (intent) is recorded using predefined categories (accident, interpersonal violence, or suspected suicide). Information on injury intent was available for 98.3% of patients in the present dataset. Subsequently, the specific mechanism of injury (e.g., fall, traffic collision, penetrating trauma) is documented. All patients primarily admitted or transferred with a severe traumatic suicide attempt from hospitals in Germany, Austria and Switzerland between 2010 and 2023 were included, provided they had an ISS ≥ 9 and were aged ≥ 10 years. No documented suicide attempts were present in younger patients. Patients who were transferred to an external hospital early (<48 h) were excluded, as no outcome data were available for these cases.

Statistics

Statistical analysis was performed using SPSS (version 29, IBM Inc., Armonk, NY, USA). Categorical variables are reported as counts and percentages. Temporal changes over the observation period were assessed using the chi-squared test for trend (linear-by-linear association test). No pairwise comparisons between individual years were performed. The analyses performed in this study are primarily descriptive and focus on temporal trends within the registry data.

The significance level was set at 0.05. Selected results were additionally presented using confidence intervals.

## 3. Results

**Figure 1:**
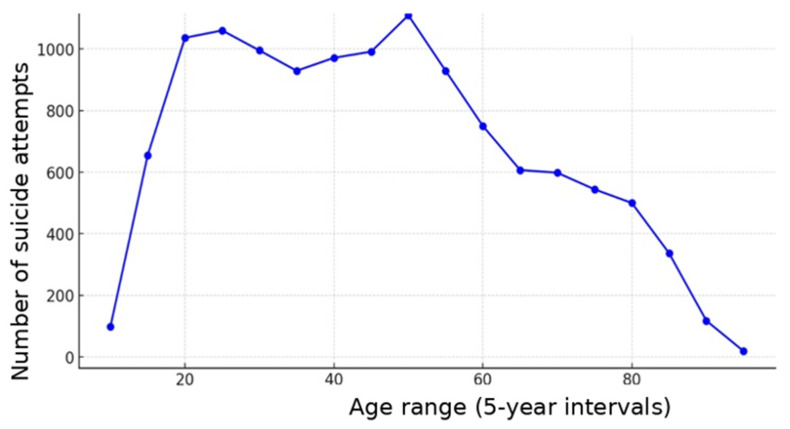
Age distribution of severe traumatic suicide attempts (5-year intervals).

A total of 265,897 patients were analyzed, of whom 12,247 (4.4%) were classified as having attempted suicide. Figure 1 illustrates the age distribution and number of severe traumatic suicide attempts over the observation period from 2010 to 2023. A clear age-dependent pattern is evident, with a marked increase beginning at approximately 15 years of age and a plateau between 20 and 55 years.

**Figure 2:**
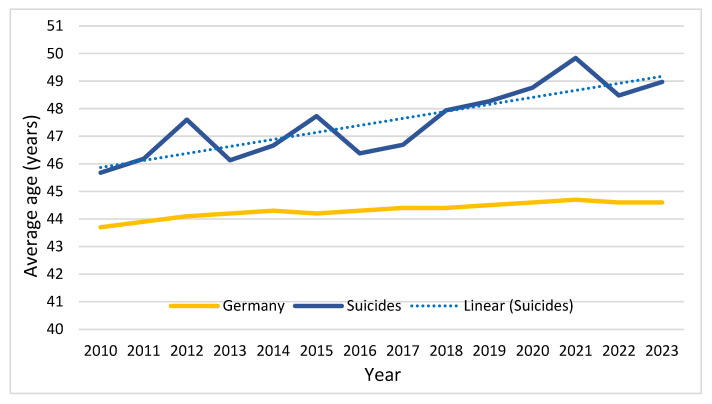
Development of the mean age of patients with suspected suicide attempts compared with the mean age of the German population between 2010 and 2023. The dashed line represents the linear trend of the mean age among suspected suicide attempts.

In the overall age distribution of the German population between 2011 and 2023, the proportions of younger age groups (<20 years and 20–40 years) remained largely stable. In contrast, the proportion of individuals aged 40–60 years declined from 31.1% in 2011 to 26.8% in 2023. During the same period, the proportion of individuals aged 60–80 years increased from 21.4% to 22.6%, while the proportion of those aged 80–100 years rose from 5.3% to 7.2% [8]. When comparing the temporal development of the mean age of patients with suspected suicide attempts to the mean age of the general population in Germany, the increase in the suicide cohort was considerably stronger (+3.3 years vs. +0.9 years). This suggests that the observed increase in older patients cannot be explained solely by demographic aging of the population but may reflect a disproportionate rise in severe traumatic suicide attempts among older individuals (Figure 2).

Across the entire study period, the predominant method of severe traumatic suicide attempt was jumping from a great height (3 m and above), accounting for 65.3%, followed by stab injuries (11.9%) and gunshot injuries (8.0%), with motor vehicle collisions involving a car (5.1%) and other traffic-related incidents (including train incidents and run-over trauma; 4.2%) occurring less frequently. Method-specific analysis from 2010 to 2023 showed a significant decrease in jumps from great height (69.2% in 2010 vs. 61.4% in 2023, *p* = 0.002) and a significant increase in penetrating injuries (18.5% in 2010 vs. 23.4% in 2023, *p* = 0.022).

**Figure 3:**
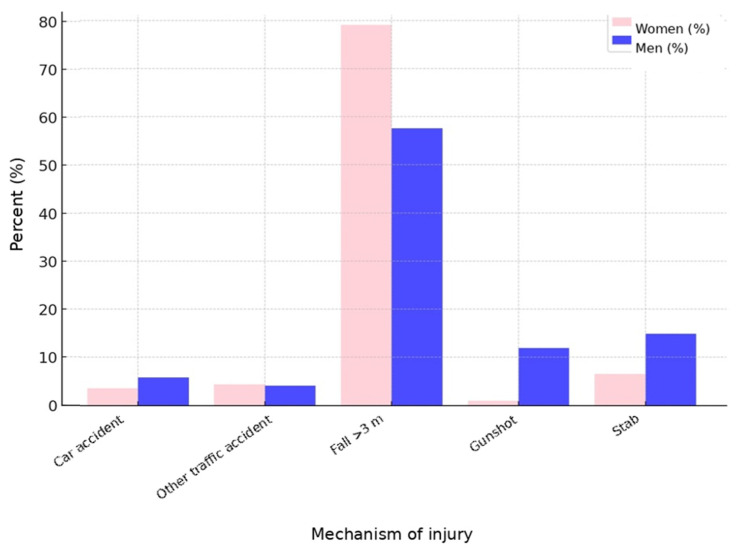
Mechanisms of severe traumatic suicide attempts in women and men.

Figure 3 depicts the mechanism of injury; jumping from a great height predominated in both men and women, while a sex-specific difference was observed in the higher proportion of penetrating injuries among men. In the present study, male patients were consistently overrepresented among severe traumatic suicide attempts, with a male-to-female ratio ranging from 1.44 to 2.17 across the observation period. Since the TraumaRegister DGU^®^ captures primarily severe traumatic injuries, the predominance of male patients may reflect the higher likelihood of men engaging in trauma-related suicide mechanisms such as penetrating injuries or high-energy trauma.

**Figure 4:**
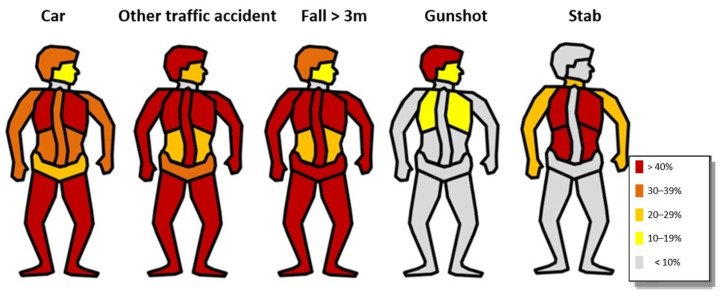
Distribution of injuries.

Distinct injury patterns were observed depending on the mechanism of injury. Figure 4 illustrates the distribution of injuries across different body regions. Injuries were categorized into nine anatomical regions according to the Abbreviated Injury Scale (AIS) and are presented for injuries with an AIS severity score ≥ 2.

Patients involved in motor vehicle collisions or other traffic-related incidents (e.g., train-related injuries or run-over trauma), as well as those who sustained falls from heights greater than 3 m, showed the typical distribution pattern of high-energy trauma, with severe injuries affecting multiple body regions.

In contrast, penetrating trauma demonstrated distinct injury patterns: head injuries predominated in gunshot wounds, whereas stab injuries were most commonly located in the thorax, neck, or upper extremities.

The highest in-hospital mortality was observed in patients with gunshot injuries (67.9%), followed by falls from >3 m (23.0%), other traffic-related incidents (22.6%), motor vehicle collisions (14.7%), and stab injuries (11.8%).

Trends in severe traumatic suicide attempts

**Figure 5:**
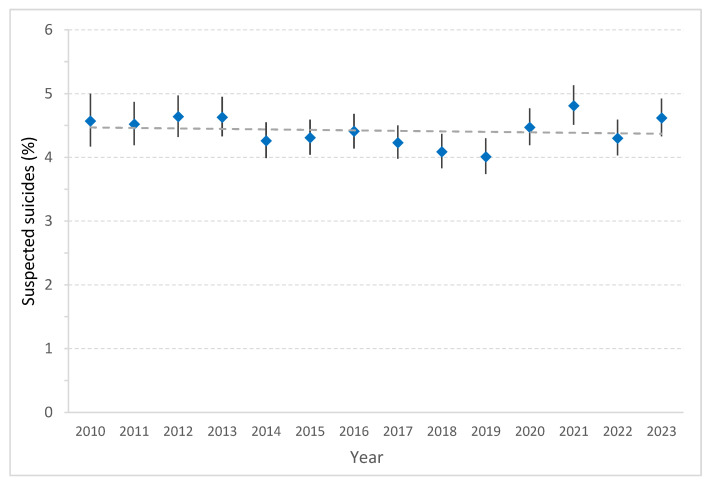
Suspected suicides (%) among all trauma patients with ISS ≥ 9 between 2010 and 2023. Points represent yearly proportions with 95% confidence intervals; the dotted line indicates the linear trend over time.

From 2010 to 2019, a significant decline in the proportion of patients with severe traumatic suicide attempts was observed, from 4.6% to 4.0% (*p* < 0.001). In 2020, a significant increase to 4.5% was noted, with comparable levels in the subsequent years (Figure 5).

Over the years, the mean age increased from 46 years in 2010 to 49 years in 2023. Table 1 presents the age trend further stratified into three age groups. The distribution of age groups changed significantly over time. Adolescents aged 10–19 years accounted for a relatively small proportion of cases throughout the study period, ranging from 5.0% to 7.2%. In contrast, the proportion of patients aged 60–99 years increased steadily from 24.4% in 2010 to 32.5% in 2023, while the proportion of patients aged 20–59 years decreased from 68.4% to 60.9%.

A chi-square test for linear-by-linear association confirmed a significant temporal trend in age group distribution (*p* < 0.001).

**Figure 6:**
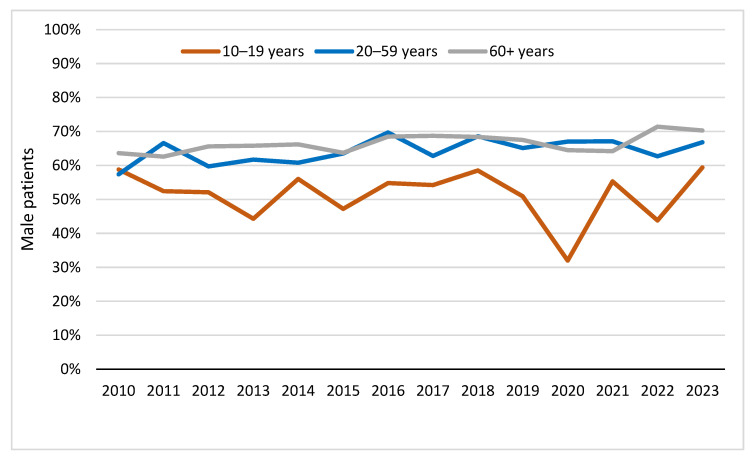
Sex distribution across age groups among patients with suspected suicide attempts between 2010 and 2023.

In an additional analysis (Figure 6), we also evaluated sex-specific trends within the adolescent subgroup. Sex-specific analyses across age groups revealed distinct patterns. While male patients predominated in the age groups 20–59 years and ≥60 years throughout the study period, the proportion of male patients was considerably lower in the adolescent group (10–19 years). In recent years, this resulted in a relatively higher proportion of female patients among adolescents compared with older age groups.

## 4. Discussion

In the present study, severe suicide attempts involving trauma-inducing mechanisms were analyzed using data from the TR-DGU. Thus, this is not an overview of all suicides, but includes cases in which individuals reached hospital alive and were recorded in the TR-DGU. The hypothesis examined was whether, under the influence of the COVID-19 pandemic, a greater number of younger patients would attempt suicide using trauma-inducing mechanisms. The results showed that, following a significant decline in severe suicide attempts involving trauma-inducing mechanisms up to 2019, a significant increase occurred in 2020, with no subsequent decrease to the previous level. The hypothesis that the COVID-19 pandemic would lead to an increase in suicide attempts among younger patients could not be confirmed in the present study. Instead, the data demonstrate a continuous aging trend among patients with severe traumatic suicide attempts. This development may partly reflect demographic changes within the general population. 

When analyzing smaller age strata, adolescents aged 10–19 years represented only a small proportion of cases within the TraumaRegister DGU^®^. In contrast to previous reports from pediatric intensive care cohorts, no clear increase in adolescent suicide attempts was observed during the pandemic years. However, a more detailed analysis suggested a relative increase among female adolescents in recent years, whereas male adolescents did not show a comparable trend. These differences from the literature may partly reflect the methodological differences between datasets, as the TraumaRegister DGU^®^ captures only severely injured trauma patients, while many suicide attempts among adolescents involve non-traumatic mechanisms such as intoxication and are therefore not represented in trauma registries.

The central event in 2020 was the onset of the COVID-19 pandemic in Germany, with lockdown and restrictions on social contacts [9]. Increasing social isolation and psychological strain during the pandemic may have been key influencing factors. The evidence regarding the impact of the pandemic on suicide presents a heterogeneous picture. In the study by Radeloff et al., no effect on suicides in the overall cohort was observed during the study period 2017–2021 [6]. Wollschläger et al. likewise found no higher number of suicides in 2020 in Rheinland-Pfalz and Emilia-Romagna compared with the years 2011–2019 [10]. By contrast, the study by Bruns et al. reported a significant increase in suicide attempts among male adolescents during the second lockdown in Germany [7]. In an analysis of data from police crime statistics and the national cause-of-death statistics of the Federal Statistical Office in Germany, Radeloff et al. demonstrated a shift from declining or stable suicide rates to increasing suicide rates, predominantly in 2022 [11]. In particular, in the age group over 60 years, the authors identified a marked trend reversal with rising suicide rates. However, the temporal coincidence with the onset of the COVID-19 pandemic suggests a possible association, although causal conclusions cannot be drawn. Other societal stressors, including economic uncertainty and geopolitical developments, may also have contributed to the observed trend. Possible explanations discussed included higher prevalence of mental illness, increased financial concerns, and geopolitical uncertainty in the context of the COVID-19 pandemic and the war in Ukraine, with downstream effects on inflation and energy supply [11].

Our study likewise demonstrated a trend reversal from declining severe suicide attempts involving trauma-inducing mechanisms up to 2019 to a significant increase in 2020, with similar levels persisting through 2023. Owing to the retrospective design, the causes of this increase cannot be proven. However, an explanatory link to the COVID-19 pandemic, which began in 2020, appears plausible. For the subsequent years up to 2023, the explanatory considerations proposed by Radeloff et al. should also be taken into account [11]. The findings of the present study should be interpreted in the context of population-based suicide statistics. While several studies reported stable suicide mortality during the COVID-19 pandemic, our analysis focuses specifically on severe traumatic suicide attempts treated in trauma centers. These datasets therefore capture different aspects of suicidal behavior. Possible explanations for the observed differences include changes in the distribution of suicide methods, variations in the severity of suicide attempts within trauma-related mechanisms, and potential changes in healthcare utilization or emergency response patterns during the pandemic period.

The age trend in both the overall cohort and patients with severe traumatic suicide attempts is increasing and may be partly explained by demographic change. This is underscored by the increase in the proportion of patients aged >70 years from 13.6% in 2010 to 19.6% in 2023. In our study, no clustering of an increase in severe traumatic suicide attempts in adolescence, as hypothesized, was observed. However, high numbers of severe traumatic suicide attempts were evident in young adulthood, remaining at a similarly high level in patients up to the age of 50 years.

The sex-specific differences identified are consistent with the literature [4]. Of note is the significant increase in penetrating injuries. Given the established knowledge that the availability of suicide methods substantially influences method choice, a critical discussion regarding the availability of penetrating weapons is warranted [12]. This is underscored by the highest mortality rate among patients with gunshot injuries. The significant decline in jumps from great height is encouraging, but from a trauma surgical perspective this remains the most common method of severe traumatic suicide attempt [13]. In clinical care, severe traumatic suicide attempts involving jumps from great height and traffic-related incidents require preparedness for severe multi-region injuries. By contrast, penetrating injuries are often isolated injuries. From a medical perspective, structural measures to prevent jumps from great height include safety barriers at high-risk locations such as bridges or railway platforms as well as restrictions on the availability of penetrating weapons are necessary. Sex-specific patterns may have implications for prevention strategies, as interventions targeting access to highly lethal methods may particularly affect male suicide attempts because of the higher male to female ratio.

The present study has several limitations. First, the TraumaRegister DGU^®^ includes only severely injured trauma patients (ISS ≥ 9) who reached hospital alive. Consequently, completed suicides occurring at the scene and patients with minor injuries are not captured in this dataset. As the classification of injury intent is based on clinical documentation, it cannot be completely excluded that some events coded as accidents may represent unrecognized suicide attempts. In addition, suicide attempts involving non-traumatic mechanisms, such as intoxication or poisoning, are not represented. Therefore, the results of this study should not be interpreted as reflecting suicide attempts in general, but rather severe traumatic injuries resulting from suicide attempts among patients surviving to hospital admission. The present analysis reflects a trauma surgical perspective on severe traumatic suicide attempts. The TraumaRegister DGU^®^ primarily captures injury mechanisms, injury severity, and clinical outcomes, but does not include detailed data on psychiatric diagnoses, neurocognitive factors, or neurobiological vulnerability patterns. These aspects are important for understanding suicidal behavior but require dedicated investigation in psychiatric or neurobiological studies.

## 5. Conclusions

The analysis of 12,247 severe suicide attempts involving trauma-inducing mechanisms highlights, in addition to the temporal association with the COVID-19 pandemic, structural challenges for the healthcare system. The significant increase in severe traumatic suicide attempts from 4.0% to 4.5% from 2020 onwards, with persistently elevated levels through 2023, underscores societal vulnerability during times of crisis. Sex-specific differences in method choice require differentiated prevention strategies. The cause of the increase in penetrating injuries (from 18.5% to 23.4%), in the context of the highest mortality rate following gunshot injuries (67.9%), remains unclear. Demographic change is reflected in the marked increase in severe traumatic suicide attempts among older patients (>70 years, from 13.6% to 19.6%) and indicates the need for age-specific prevention approaches. Survivors of severe suicide attempts represent a particularly vulnerable group. Therefore, structured interdisciplinary care pathways including psychiatric assessment and psychosocial support are essential after the acute trauma treatment.

## Figures and Tables

**Table 1:** Age groups of patients who attempted suicide, and the relative distribution across three age groups per year in the period 2010–2023.

Year	10–19 Years Old	20–59 Years Old	60–99 Years Old
2010	7.2%	68.4%	24.4%
2011	6.2%	67.9%	25.9%
2012	6.0%	67.1%	26.9%
2013	6.8%	67.3%	25.9%
2014	5.5%	68.8%	25.6%
2015	5.4%	67.6%	26.9%
2016	6.0%	69.1%	24.9%
2017	7.0%	66.2%	26.8%
2018	5.8%	64.8%	29.4%
2019	6.6%	63.6%	29.8%
2020	5.4%	63.3%	31.2%
2021	5.0%	61.7%	33.3%
2022	7.1%	61.7%	31.3%
2023	6.6%	60.9%	32.5%

## Data Availability

Data available on request.

## Abbreviations

The following abbreviations are used in this manuscript:

ISS	Injury Severity Score
TR	TraumaRegister
